# A novel family VIII carboxylesterase hydrolysing third- and fourth-generation cephalosporins

**DOI:** 10.1186/s40064-016-2172-y

**Published:** 2016-04-26

**Authors:** Jeong Ho Jeon, Hyun Sook Lee, Jung Hun Lee, Bon-Sung Koo, Chang-Muk Lee, Sang Hee Lee, Sung Gyun Kang, Jung-Hyun Lee

**Affiliations:** Marine Biotechnology Research Division, Korea Institute of Ocean Science and Technology, Ansan, 15627 Republic of Korea; Department of Marine Biotechnology, University of Science and Technology, Daejeon, 34113 Republic of Korea; National Leading Research Laboratory of Drug Resistance Proteomics, Department of Biological Sciences, Myongji University, 116 Myongjiro, Yongin, Gyeonggido, 17058 Republic of Korea; Department of Agricultural Biotechnology, National Academy of Agricultural Science, RDA, Jeonju, 54875 Republic of Korea

**Keywords:** Metagenome, β-Lactamase, Carboxylesterase, Extended-spectrum cephalosporins

## Abstract

A metagenomic library was constructed from a soil sample of spindle tree-rhizosphere. From this library, one clone with esterase activity was selected. The sequence analysis revealed an open reading frame (EstSTR1) encoded protein of 390 amino acids. EstSTR1 is a family VIII carboxylesterase and retains the S-X-X-K motif conserved in both family VIII carboxylesterases and class C β-lactamases. The *estSTR1* gene was overexpressed in *E*. *coli* and the recombinant protein was purified by purified by metal chelating affinity chromatography and size-exclusion chromatography. EstSTR1 hydrolysed *p*-nitrophenyl esters, exhibited the highest activity toward *p*-nitrophenyl butyrate. Furthermore, EstSTR1 could hydrolyse third- and fourth-generation cephalosporins (cefotaxime and cefepime) as well as first-generation cephalosporin (cephalothin). Site-directed mutagenesis studies revealed that a catalytic residue, Ser71, of EstSTR1 plays an essential role in hydrolysing both antibiotics and *p*-nitrophenyl esters. We demonstrate that a metagenome-derived carboxylesterase displays β-lactam-hydrolysing activities toward third- and fourth-generation cephalosporins.

**Electronic supplementary material:**

The online version of this article (doi:10.1186/s40064-016-2172-y) contains supplementary material, which is available to authorized users.

## Background

Carboxylesterases (EC3.1.1.1) hydrolyse emulsified esters of short-chain carboxylic acids, retaining a characteristic α/β hydrolase fold with the G-X-S-X-G motif in the active site which contains a conserved serine residue (Bornscheuer 2002; Nardini and Dijkstra 1999). Based on conserved sequence motifs and biological properties, microbial carboxylesterases have been classified into eight families (Arpigny and Jaeger 1999). Particularly, the primary sequences of family VIII carboxylesterases with a conserved S-X-X-K motif instead of the G-X-S-X-G motif of canonical carboxylesterases are similar to those of class C β-lactamases (Arpigny and Jaeger 1999). A previous report (Wagner et al. 2002) revealed that the overall structure of EstB from *Burkholderia gladioli*, composed of a mixed α/β domain and a small helical domain, was similar to that of class C β-lactamases.

Functional metagenomic screening, in which shotgun-cloned DNA fragments are selected for survival to antibiotic exposure, have been increasingly applied to the characterisation of many antibiotic resistance reservoirs. In recent study, a new class A β-lactamase derived from a polluted river metagenome library has been identified by functional screening (Vercammen et al. 2013). These experiments have demonstrated that antibiotic resistance genes are highly diverse and widely distributed, frequently bearing little to no similarity to known sequences (Pehrsson et al. 2013). The relationship between serine-β-lactamases and family VIII carboxylesterases has been surprisingly evidenced by the metagenomic approach of screening ester-hydrolysing activities that perform unrelated functions to previously identified resistance genes. New family VIII carboxylesterases that originated from metagenomic libraries of various environmental samples (Elend et al. 2006; Rashamuse et al. 2009; Kim et al. 2010; Jeon et al. 2011; Yu et al. 2011; Mokoena et al. 2013), including EstC (Rashamuse et al. 2009), EstM-N1 (Yu et al. 2011), EstM-N2 (Yu et al. 2011), and Est22 (Mokoena et al. 2013), exhibited hydrolysing activity for nitrocefin, but did not display β-lactamase activity for β-lactam antibiotics. Recently, the family VIII carboxylesterase, EstU1, which was selected from a soil metagenomic library by screening for esterase activity showed a bona fide hydrolysing activity toward both the ester bond of *p*-nitrophenyl esters and the amide bond of β-lactams, apparently utilising the same active site residues for both reactions, as supported by site-directed mutagenesis, confirming the functional relationship between family VIII carboxylesterases and β-lactamases (Jeon et al. 2011).

In this study, we report a novel family VIII carboxylesterase identified from a soil metagenomic library. EstSTR1 display an esterase activity for *p*-nitrophenyl esters. In particular, EstSTR1 exhibits a broad range of β-lactam hydrolytic activities for first-generation cephalosporin (cephalothin), third-generation cephalosporin (cefotaxime), and fourth-generation cephalosporin (cefepime). Here, we demonstrate the functional characterisation of EstSTR1, as well as site-directed mutagenesis and its structural discussion to understand the mechanism of EstSTR1.

## Methods

### Strains, metagenomics library construction, and screening lipolytic activity

*E. coli* DH5α and BL21(DE3) were used for all cloning and expression experiments, respectively. A soil sample (35°52′N, 127°3′E) was collected from the Korea Expressway Corporation Arboretum in Jeonju City, South Korea. Soil DNA was prepared by directed DNA extraction and purification as previously described (Kim et al. 2007). The metagenomic cosmid library was constructed according to the method of Yun et al. (2005). Two-step DNA purification was applied to remove humic compounds present in the soil DNAs using pulsed-field gel electrophoresis (PFGE) (CHEF, BioRad). To remove humic compounds in DNA extracted from soil sample, 1 % low melting point agarose was prepared and crude DNA was fractionated by PFGE under a 4 V cm^−1^ electrical field at 14 °C for 12 h. A gel containing 100–190 kb of DNA was purified by agarase (1 U per 100 mg slice, Takara, Japan). The isolated DNA was partially digested by *Sau3A*I (0.05 U μl^−1^ of DNA, 37 °C for 1 h), and then the digested DNA was fractionated by PFGE. A gen containing approximately 40-kb lengths DNA was again purified by agarase. The 40-kb DNA was ligated into a pSuperCosI (Stratagene, La Jolla, CA) and packaged using MaxPlax Lambda Packaging Extracts (Epicentre, Madison, WI). For screening esterase activity, the transformants were plated on Luria–Bertani (LB) agar plates with chloramphenicol (12.5 μg ml^−1^) and tributyrin (1 %). After incubation at 37 °C for 1 day, the plates were subsequently incubated at 4 °C for a week. Candidate colonies were selected based on the presence of clear zone on the plate.

### Subcloning and sequence analysis

Positive colony (pCosSTR1) with a clear halo was selected on the plate. To identify the gene encoding esterase active, the subclone library was constructed by method as described by Jeon et al. (2011). The transformants with esterase activity were selected by the presence of the clear halo zone on LB agar plates containing 100 μg ml^−1^ ampicillin and 1 % tributyrin. DNA sequencing of the subclone with esterase activity was performed with an ABI3100 (PE Applied Biosystems, Foster City, CA, USA) and assembled using the Vector NTI suite 7 software package (InforMax, North Bethesda, MD, USA). The open reading frames (ORFs) and sequence homology searches in the complete assembled sequence were analysed by the ORF finder and BlastX search provided by the National Center for Biotechnology Information (NCBI) (Altschul et al. 1997).

### Multiple alignment and phylogenetic analysis

Multiple sequence alignments were carried out by the ClustalW program (Thompson et al. 1994) for the protein sequences. To compare the amino acid sequences of EstSTR1 and EstU1, we performed sequence alignment using the Expresso program on the T-Coffee Server (Notredame et al. 2000), found at the ExPASy web site (http://tcoffee.vital-it.ch/apps/tcoffee/index.html). Expresso is able to combine sequence information with protein structural information. Molecular Evolutionary Genetics Analysis 4.1 software (MEGA, version 4.1) (Tamura et al. 2007) was used to make the phylogenetic tree using a neighbour-joining method (Saitou and Nei 1987).

### Expression and purification of recombinant EstSTR1

The *estSTR1* gene was amplified by PCR the following primers: STR1-F (5′-GAGACCCCATATGAGCACCGGGATCGAAATTCAAG-3′) and STR1-R (5′-CTATCTCGAGGCTGTTCGGCAGGCAGCGATAC-3′). *Nde*I and *Xho*I sites for cloning are underlined. The PCR product (1173 bp) for the *estSTR1* gene was cloned into pET-24a (+) vector containing the T7 polymerase promoter system (Novagen, Madison, Wisconsin, USA), and then the recombinant plasmid was introduced into *E. coli* BL21(DE3) cells. When *E. coli* BL21(DE3) cell harbouring pET-24a(+)/His_6_-EstSTR1 reached approximately 0.6 at 600 nm, 1 mM IPTG (isopropyl β-d-1-thiogalactopyranoside) was added to induce expression. The cells were harvested by centrifugation (5000×*g*, 4 °C, 20 min) after induction at 25 °C for 10 h, resuspended in 10 mL of buffer A (50 mM Tris–HCl pH 8.0, 10 % glycerol, 100 mM KCl). The solution was vortexed, and then sonicated for 20 min. To obtain the soluble protein, the crude lysate disrupted by sonication was centrifuged (15,000×*g*, 4 °C, 60 min). To purify EstSTR1 with the His_6_ tag, the soluble proteins were loaded onto a column of TALON^®^ metal affinity resin (BD Biosciences Clontech, Palo Alto, CA, USA) and washed with buffer B (50 mM Tris–HCl pH 8.0, 10 mM imidazole, 10 % glycerol, 100 mM KCl). The bound EstSTR1 was eluted with buffer C (50 mM Tris–HCl pH 8.0, 300 mM imidazole, 10 % glycerol, 100 mM KCl). For further purification, size exclusion chromatography was performed. Eluted sample was purified using Superdex-75 (16/60) column (GE Healthcare, Piscataway, NJ, USA) equilibrated and run using buffer D (150 mM NaCl, 20 mM Tris–HCl pH, 7.8).

### Characterizations of EstSTR1 for pNP esters

Enzyme activity was determined by colorimetric method measuring released *p*-nitrophenol from *p*-nitrophenyl (*p*NP) esters (Sigma, St. Louis, MO, USA) at 405 nm. Esterase activity was measured by reaction mixture with 1 mM *p*-nitrophenyl esters in 50 mM Tris–HCl (pH 8.0) containing 1 % (v/v) acetonitrile at 35 °C. The substrate specificity of enzyme was determined in the presence of 1 mM of *p*-nitrophenyl esters with different aliphatic side chains: acetate (C2), butyrate (C4), hexanoate (C6), octanoate (C8), decanoate (C10), laurate (C12), myristate (C14), palmitate (C16), and stearate (C18). The optimum temperature of enzyme was determined at temperatures ranging from 5 to 70 °C using *p*-nitrophenyl butyrate as a substrate in 50 mM Tris–HCl buffer (pH 8.0). For optimization of the pH of enzyme, enzyme activity was measured for a pH range of 4.0–10.0. The buffers used were 50 mM sodium acetate (pH 4.0–6.0), 50 mM sodium phosphate (pH 6.0–7.5), 50 mM Tris–HCl (pH 7.5–8.5), and 50 mM CHES (pH 8.5–10.0).

### β-Lactamase assay of EstSTR1

Antibiotics (cephalothin, cefoxitin, cefotaxime, and cefepime) were obtained from Sigma-Aldrich (St. Louis, MO, USA). The chemical structures of cephalosporins (cephalothin, cefoxitin, cefotaxime, and cefepime) are shown in the Additional file 1: Figure S1. The hydrolysing activity of EstSTR1 for cephalosporins was measured by the paper disc method as previously described (Jeon et al. 2011). The enzyme (330 μM) was incubated with antibiotic substrates (3 mM cephalothin, 1 mM cefoxitin, 1 mM cefotaxime, and 1 mM cefepime) in 50 mM Tris–HCl (pH 8.0) for 2 h at 35 °C, and then reaction mixtures were loaded onto small paper discs. After 8 h incubation at 37 °C, the diameters of the inhibition zones around the small paper discs were recorded. For comparison of β-lactam hydrolysing activity, a negative control containing antibiotics without enzyme and a positive control containing antibiotics and the CMY-10, a plasmid-encoded class C extended-spectrum β-lactamase (ESBL) from *Enterobacter aerogenes* (Kim et al. 2006), were used.

### Determination of kinetic parameters

The kinetic parameters (k_cat_ and K_m_) of EstSRT1 were obtained by measuring the absorbance variation using the molecular extinction coefficient of each substrate: *p*-nitrophenyl butyrate (Δε_405_ = 13,500 M^−1^ cm^−1^), cephalothin (Δε_262_ = −7660 M^−1^ cm^−1^), cefotaxime (Δε_264_ = −7250 M^−1^ cm^−1^), and cefepime (Δε_267_ = −9120 M^−1^ cm^−1^). The assay for *p*-nitrophenyl butyrate was conducted in 50 mM CHES (pH 9.0) containing (approximately 2.43 nM) and substrates (10–600 μM). The assays for β-lactam substrates were conducted in 10 mM MES buffers (pH 6.8) with enzymes (396 μM), substrates (10–500 μM), and bovine serum albumin (20 μg ml^−1^). Steady-state kinetic constants were determined by fitting the initial rates (in triplicate) directly to the Henri-Michaelis–Menten equation using non-linear regression with the program *DYNAFIT* (Kuzmic 1996). When K_m_ values for β-lactam substrates were too low to be determined, the values were determined as competitive inhibition constants, K_i_, in the presence of a reporter substrate (100 μM cephalothin), and K_i_ values were calculated as previously described (Galleni and Frere 1988; De Meester et al. 1987).

### Site-directed mutagenesis of EstSTR1

A site-directed change to alanine (S71A) was made using the Stratagene Quik Change kit (La Jolla, CA, USA). The primers (S71A-F and S71A-R) designed to introduce the S71A substitution were as follows: S71A-F (5′-CGCTCATCAATACCTATGCGACCACCAAGGGCATGG-3′) and S71A-R (5′-CCATGCCCTTGGTGGTCGCATAGGTATTGATGAGCG-3′). The sequence corresponding to the mutated codons are underlined. The catalytic activity of the variant was tested and compared with that of the wild-type enzyme.

### Nucleotide sequence accession number

The nucleotide sequence of EstSTR1 has been deposited in the GenBank database under the accession number KJ530984.

## Results

### Metagenomic library screening and sequence analysis

**Fig. 1:**
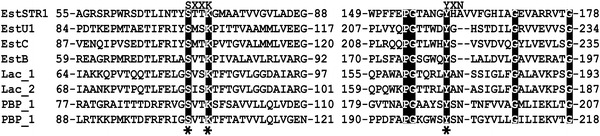
Conserved sequence blocks from a multiple sequence alignment of EstSTR1 and related family VIII carboxylesterases, class C β-lactamases, and penicillin-binding proteins (PBPs). Family VIII carboxylesterases are represented by EstU1 (uncultured bacterium, JF791800), EstC (uncultured bacterium, ACH88047), and EstB (*Burkholderia gladioli*, AAF59826). Class C β-lactamases are represented by Lac-1 (*Escherichia coli*, AAA23441) and Lac-2 (*Enterobacter cloacae*, P05364) and penicillin-binding proteins are represented by PBP-1 (*Streptomyces* sp. R61, P15555) and PBP-2 (*Bacillus cereus*, CAA09676). *Asterisks* indicate conserved active site residues. Identical residues are shown as *white letters* on a dark background

**Fig. 2:**
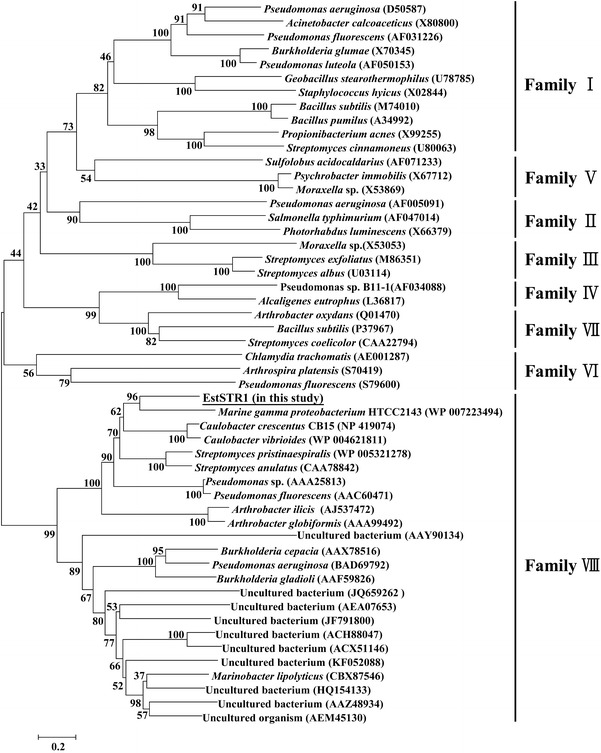
Phylogenetic relationship of EstSTR1 with the lipolytic enzymes including family VIII carboxylesterases. The tree was constructed using the MEGA 4.1 program with the neighbor-joining algorithm. Only bootstrap values higher than 50 % are shown. *Bar* 0.2 substitutions per amino acid site

A cosmid metagenomic library consisting of 7968 clones was constructed using high molecular weight DNA extracted from a soil sample of spindle tree-rhizosphere, taken near the Korea Expressway Corporation Arboretum in South Korea. The insert sizes for the cosmid clones ranged from 35 to 40 kb, with non-redundant patterns. The entire library was screened for lipolytic activity on 1 % tributyrin plates. One cosmid clone (36 kb insert size) forming a clear halo zone on the plate was selected for further analysis. To characterise the gene exhibiting esterase activity, the subcloning experiment with a pUC118/HincII/BAP plasmid was performed and subclone (pUCSTR1) with a short insert of 1825 bp was found. The sequence analysis of the pUCSTR1 insert DNA showed the presence of one open reading frame (ORF) of 1173 bp (*estSTR1*), encoding a polypeptide of 390 amino acids. Primary sequence analysis of EstSTR1 indicated that it was similar to a putative esterase (WP_007223494) from *Marine gamma proteobacterium* HTCC2143 (47 % identity), class C β-lactamase (WP_004621811) from *Caulobacter vibrioides* (47 % identity), and β-lactamase (WP_005321278) from *Streptomyces pristinaespiralis* (46 % identity). Multiple sequence alignment of EstSTR1 and its homologs showed that the S-X-X-K motif is well conserved in family VIII carboxylesterases (Jeon et al. 2011; Kim et al. 2010; Rashamuse et al. 2009; Elend et al. 2006), class C β-lactamases (Knox et al. 1996), and penicillin-binding proteins (PBPs) (Joris et al. 1988) (Fig. 1). Based on the esterase/lipase classification system (families I–VIII) proposed by Arpigny and Jaeger (1999), the phylogenetic relationship was analysed. The phylogenetic analysis showed that EstSTR1 was grouped with family VIII carboxylesterases (Fig. 2).

### Purification and characterisation of EstSTR1

**Fig. 3:**
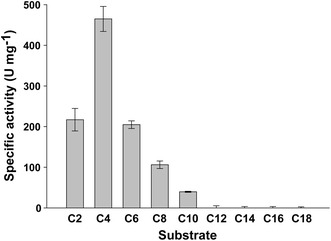
Substrate preference of the purified EstSTR1 toward *p*-nitrophenyl esters. Nine *p*-nitrophenyl esters: *C2* *p*-nitrophenyl acetate, *C4* *p*-nitrophenyl butyrate, *C6* *p*-nitrophenyl hexanoate, *C8* *p*-nitrophenyl octanoate, *C10* *p*-nitrophenyl decanoate, *C12* *p*-nitrophenyl laurate, *C14* *p*-nitrophenyl myristate, *C16* *p*-nitrophenyl palmitate, *C18* *p*-nitrophenyl stearate. *Error bars* indicate standard deviations (*n* = 3)

To invest the biochemical properties of EstSTR1, the *estSTR1* gene ligated into pET-24a(+) was overexpressed in *E. coli*. SDS-PAGE analysis of purified EstSTR1 showed a single protein band which correlated well with the theoretical mass (42 kDa) of EstSTR1 (Additional file 1: Figure S2). The hydrolytic activity of purified recombinant EstSTR1 for *p*-nitrophenyl esters (C2–C18) was investigated. EstSTR1 showed substrate preference for a wide range of substrates (C2 to C10), and *p*-nitrophenyl butyrate (C4) was most rapidly hydrolysed (Fig. 3). However, no enzyme activity was detected for *p*-nitrophenyl esters (C12–C18) with a longer chain (Fig. 3). The optimum activity of EstSTR1 was measured at a temperature range of 5–70 °C and pH range of 6.0 and 10.0. The enzyme showed a temperature optimum at 40 °C (Additional file 1: Figure S3A). The optimum activity occurred at an alkaline pH in the range of pH 9–10, threefold higher than the activity at pH 8.0, indicating that EstSTR1 is an alkaline esterase (Additional file 1: Figure S3B).

### Determination of β-lactamase activity

**Fig. 4:**
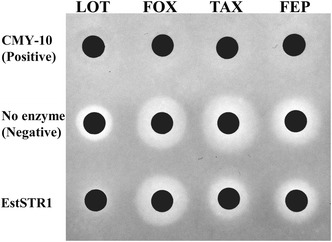
Disc diffusion assay for confirming the hydrolysis of antibiotics by EstSTR1. This assay was performed by incubating purified EstSTR1 with antibiotics, including 3 mM cephalothin, 1 mM cefoxitin, 1 mM cefotaxime, and 1 mM cefepime in 50 mM Tris–HCl (pH 8.0) for 2 h at 35 °C. The reaction mixtures were adsorbed onto a paper disk and placed on agar seeded with *E. coli* BL21(DE3). The diameters of the inhibition zones around the discs appeared after an 8 h at 37 °C. The negative controls were samples containing antibiotics only and the positive controls were reaction mixtures containing CMY-10 and antibiotics. Antibiotics: *LOT* cephalothin, *FOX* cefoxitin, *TAX* cefotaxime, *FEP* cefepime

Previously, we reported that a metagenome derived esterase, a family VIII carboxylesterase (EstU1) could hydrolyse first-generation cephalosporins (cephaloridine, cephalothin, and cefazolin) (Jeon et al. 2011). The crystal structure of cephalothin complex of EstU1 revealed an acyl-enzyme intermediate in which Ser100 (the nucleophile) in first motif of EstU1 is covalently linked to the carbonyl carbon of the hydrolysed β-lactam ring of cephalothin (Cha et al. 2013). Though the amino acid sequence of EstSTR1 showed low similarity to EstU1 from uncultured bacterium (15 % identity), three active site residues (S100, K103, and Y218) essential for the β-lactam hydrolytic activity in EstU1 could be found in EstSTR1 (Fig. 1). For these reasons, we examined whether EstSTR1 could hydrolyse various β-lactam antibiotics, cephalothin (first-generation cephalosporins), cefoxitin (second-generation cephalosporin), cefotaxime (third-generation cephalosporins), and cefepime (fourth-generation cephalosporin). Compared with that of the negative control, the diameters of the inhibition zones around the discs containing cephalothin, cefotaxime, and cefepime incubated with EstSTR1 were decreased from 27 to 22 mm in cefotaxime and from 26 to 23 mm in cefepime (Fig. 4). The result implies that the antibiotic efficacies of cephalothin, cefotaxime, and cefepime are affected by EstSTR1 activity. However, EstSTR1 did not appear to change the antibiotic efficacy of cefoxitin because the size alteration of the clear zone around paper disc did not show compared with that of the negative control. Thus, EstSTR1 displays β-lactam hydrolytic activity for cephalothin, cefotaxime, and cefepime except for cefoxitin.

### Determination of kinetic parameters

The kinetic parameters of EstSTR1 for cephalosporins (cephalothin, cefotaxime, and cefepime) and *p*-nitrophenyl butyrate were investigated. The K_m_ and k_cat_ values for *p*-nitrophenyl butyrate were approximately 10- and 20-fold higher than those of EstU1 (6.03 μM and 15.72 s^−1^) (Jeon et al. 2011), respectively (Table 1). Kinetic analysis of EstSTR1 revealed that this enzyme has a hydrolysing activity for cephalothin, cefotaxime, and cefepime (Table 1). The catalytic efficiencies of EstSRT1 for cephalothin, cefotaxime, and cefepime were similar to that of EstU1 for cefazolin (3.035 M^−1^ s^−1^) (Jeon et al. 2011) (Table 1). Although catalytic efficiencies of EstSTR1 for β-lactams were very low level, catalytic efficiency of β-lactamase I (4 M^−1^ s^−1^) from *Bacillus cereus* for a β-lactam (Martin Villacorta et al. 1991) is similar to the level of the EstSTR1.

**Table 1:** Kinetic parameters for hydrolysis of *p*-nitrophenyl butyrate and cephalosporins (cephalothin, cefotaxime, and cefepime) by EstSTR1 and its mutant enzyme (S71A)

Substrates	k _cat_ (s^−1^)		K_m_ (μM)		k _cat_/K_m_ (M^−1^ s^−1^)	
	Wild	S71A	Wild	S71A	Wild	S71A
*p*-nitrophenyl butyrate	297.12 ± 5.90	nh	65.62 ± 5.92	nh	4.52 ± 0.001 × 10^6^	nh
Cephalothin	4.63 ± 0.03 × 10^−5^	nh	16.58 ± 0.18	nh	2.78 ± 0.03	nh
Cefotaxime	1.57 ± 0.04 × 10^−6^	nh	0.90 ± 0.004^a^	nh	1.67 ± 0.01	nh
Cefepime	1.63 ± 0.03 × 10^−6^	nh	0.19 ± 0.003^a^	nh	8.48 ± 0.01	nh

*nh* not hydrolysed

^a^Determined *K*_i_ values

### Site-directed mutagenesis

Because EstSTR1 retains the catalytic (or nucleophilic) serine (Ser71) correlated with the Ser100 of EstU1, we wondered whether a single nucleophile (Ser71) was involved or whether the enzyme harbored two active sites. We performed site-directed mutagenesis to confirm the effect of substitution of a single serine on both activities of EstSTR1. The nucleophilic serine (Ser71) in EstSTR1 was mutated into an alanine residue (Ala71) and the mutant protein (S71A) was purified and characterised, compared with the wild-type EstSTR1. The mutant protein (S71A) was inactive for cephalosporins and *p*-nitrophenyl butyrate (Table 1). This result suggests that the serine residue is essential for both activities.

## Discussion

In this study, a novel family VIII carboxylesterase, designated as EstSTR1, was identified by active screening of a metagenomic library constructed from a soil sample of tree-rhizosphere collected near in the Korea Expressway Corporation Arboretum in South Korea. EstU1 and EstSTR1 were found within two soil functional metagenomes of really a few clones (<10,000), which means that the retrieved genes are likely to be highly prevalent in soil. The amino acid sequence of EstSTR1 showed that the S-X-X-K motif encompassing the nucleophilic serine residue of serine-β-lactamases was conserved and EstSTR1 was grouped together with the family VIII carboxylesterases. The β-lactam hydrolytic activities of EstSTR1 for first-generation cephalosporin (cephalothin), third-generation cephalosporin (cefotaxime), and fourth-generation cephalosporin (cefepime) were clearly demonstrated by the disc diffusion assay and kinetics as well.

A previous report (Cha et al. 2013) revealed that the structure of EstU1 had a β-lactamase-like modular architecture and that the active site residues (Ser100, Lys103, and Tyr218) of EstU1 played an important role in hydrolysing both ester and amide bonds (Cha et al. 2013; Jeon et al. 2011). The corresponding active site residues, Ser71, Lys74, and Tyr160, could be predicted in EstSTR1 and site-directed mutagenesis of the EstSTR1 serine residue demonstrated that this residue was crucial for both the esterase and β-lactamase activities. In addition, the active site in EstU1 was divided into R1 and R2 subsites. The R1 subsite is defined by the R1 segment, the β8–β9 turn, and the Ω-loop. The R2 subsite is surrounded by the R2’ segment, the R2 segment, and β8, with the nucleophilic serine. The R1 subsite accommodates the R1 side-chain of cephalosporins and the R2 subsite represents the opposite region interacting with the R2 side-chain of cephalosporins (Kim et al. 2006). EstU1 only hydrolysed first-generation cephalosporins (e.g., cephalothin, cephaloridine, and cefazolin); however, EstSTR1 hydrolysed third- and fourth-generation cephalosporins as well as first-generation cephalosporin. Cefotaxime and cefepime have the R1 side chain larger than that of cephalothin (Additional file 1: Figure S1). This result implies that the R1 subsite of EstSTR1 may be wider than that of EstU1. We confirmed variations in the Ω-loop and R1 segment in the R1 subsite via sequence alignment of EstSTR1 and EstU1 (Additional file 1: Figure S4). Therefore, these results suggest that variation of the R1 subsite in EstSTR1 allows the active site of EstSTR1 to access substrates (cefotaxime and cefepime), resulting in the efficient acylation.

The kinetic study of EstSTR1 also indicated that EstSTR1 showed weak β-lactamase activity for the antibiotic substrates, cephalothin, cefotaxime, and cefepime. A hydrolase mechanism for antibiotics has recently been demonstrated from the crystal structure of cephalothin complex of EstU1. A two-strep hydrolysis process composed of acylation and deacylation steps, mediated by the nucleophilic serine has been proposed in family VIII carboxylesterases and serine-β-lactamases. The weak hydrolytic activity of EstU1 for β-lactam antibiotics likely indicates that the active site of EstU1 cannot show the efficient deacylation reaction. The EstSTR1 deacylation efficiency on β-lactam antibiotics may therefore be lower than that of typical extended-spectrum β-lactamase, lowering the overall hydrolysis efficiency.

## Conclusion

One gene screened from a soil metagenomic library was identified as having an S-X-X-S motif found in family VIII carboxylesterases and serine-β-lactamases. The purified EstSTR1 protein showed hydrolysing activity for *p*-nitrophenyl esters and antibiotic substrates such as cephalothin, cefotaxime, and cefepime. Similar to EstU1, EstSTR1 contains only the active site residues (Ser100, Lys103, and Tyr218) responsible for the hydrolysing activity toward both β-lactam antibiotics and *p*-nitrophenyl esters. EstSTR1 is the first esterase that hydrolyses third-generation cephalosporin (cefotaxime) and fourth-generation cephalosporin (cefepime). To elucidate the detailed catalytic mechanism of EstSTR1, further studies will be required, including the crystallographic determination of EstSTR1’s structure.

## Additional file

10.1186/s40064-016-2172-y Chemical structures of cephalothin (A), cefoxitin (B), cefotaxime (C), and cefepime (D). The R1 and R2 side-chains located at C7 and C3 position of the β-lactam nucleus are labeled; **Figure S2.** SDS-PAGE of the purified EstSTR1 protein.M, Molecular size markers; T, whole-cell extracts; S, soluble fraction; I, insoluble fraction; P1, EstU1 purified by Ni-NTA column; P2, EstU1 purified by Superdex 75 gel filtration column. The purified EstSTR1 corresponded to a molecular mass of approximately 42 kDa and is indicated by arrow; **Figure S3.** Effects of temperature (A) and pH (B) on the activity of EstSTR1. Enzyme activity was measured using *p*-nitrophenyl butyrate as a substrate at various temperatures. The buffers used were 50 mM sodium acetate buffer (closed circles; pH 4.0 to 6.0), 50 mM sodium phosphate buffer (open circles; pH 6.0 to 7.5), 50 mM Tris–HCl buffer (closed triangles; pH 7.5 to 8.5), and 50 mM CHES buffer (open triangles; pH 8.5 to 10.0). The highest value of each enzyme activity was set at 100%; **Figure S4.** Sequence alignment of EstSTR1 with EstU1 which is Family VIII carboxylesterase. R1 segment, Ω-loop, and β8-β9 hairpin regions are indicated with red, blue, and orange boxes, respectively.
