# Supplementary material for: A novel family VIII carboxylesterase hydrolysing third- and fourth-generation cephalosporins
Source: Springerplus. 2016 Apr 26;5:525. doi: 10.1186/s40064-016-2172-y (PMC4844572; doi:10.1186/s40064-016-2172-y)
Supplement: Supplementary file 1 — 10.1186/s40064-016-2172-y Chemical structures of cephalothin (A), cefoxitin (B), cefotaxime (C), and cefepime (D). The R1 and R2 side-chains located at C7 and C3 position of the β-lactam nucleus are labeled; Figure S2. SDS-PAGE of the purified EstSTR1 protein.M, Molecular size markers; T, whole-cell extracts; S, soluble fraction; I, insoluble fraction; P1, EstU1 purified by Ni-NTA column; P2, EstU1 purified by Superdex 75 gel filtration column. The purified EstSTR1 corresponded to a molecular mass of approximately 42 kDa and is indicated by arrow; Figure S3. Effects of temperature (A) and pH (B) on the activity of EstSTR1. Enzyme activity was measured using p-nitrophenyl butyrate as a substrate at various temperatures. The buffers used were 50 mM sodium acetate buffer (closed circles; pH 4.0 to 6.0), 50 mM sodium phosphate buffer (open circles; pH 6.0 to 7.5), 50 mM Tris–HCl buffer (closed triangles; pH 7.5 to 8.5), and 50 mM CHES buffer (open triangles; pH 8.5 to 10.0). The highest value of each enzyme activity was set at 100%; Figure S4. Sequence alignment of EstSTR1 with EstU1 which is Family VIII carboxylesterase. R1 segment, Ω-loop, and β8-β9 hairpin regions are indicated with red, blue, and orange boxes, respectively. [file 40064_2016_2172_MOESM1_ESM.docx]

**SpringerPlus**

**Jeong Ho Jeon ^1,3^, Hyun Sook Lee ^1,2^, Jung Hun Lee^3^, Bon-Sung Koo^4^, Chang-Muk Lee^4^, Sang Hee Lee ^3*^, Sung Gyun Kang ^1,2*^ and Jung-Hyun Lee ^1,2*^**

^1^ Marine Biotechnology Research Division, Korea Institute of Ocean Science and Technology, Ansan, Republic of Korea

^2^ Department of Marine Biotechnology, University of Science and Technology, Daejeon, Republic of Korea

^3^ National Leading Research Laboratory of Drug Resistance Proteomics, Department of Biological Sciences, Myongji University, Yongin, Republic of Korea

^4^ Department of Functional Materials Development, National Academy of Agricultural Science, RDA, Suwon 441-707, Republic of Korea

**^*^**Correspondence: sangheelee@mju.ac.kr; sgkang@kiost.ac; jlee@kordi.re.k


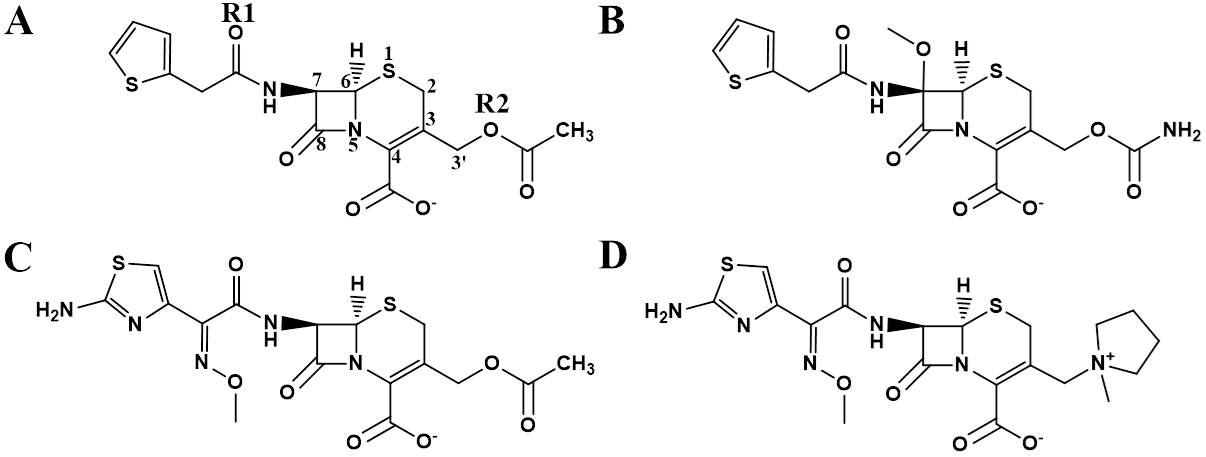


**Figure S1.** Chemical structures of cephalothin (**A**), cefoxitin (**B**), cefotaxime (**C**), and cefepime (**D**). The R1 and R2 side-chains located at C7 and C3 position of the β-lactam nucleus are labeled.


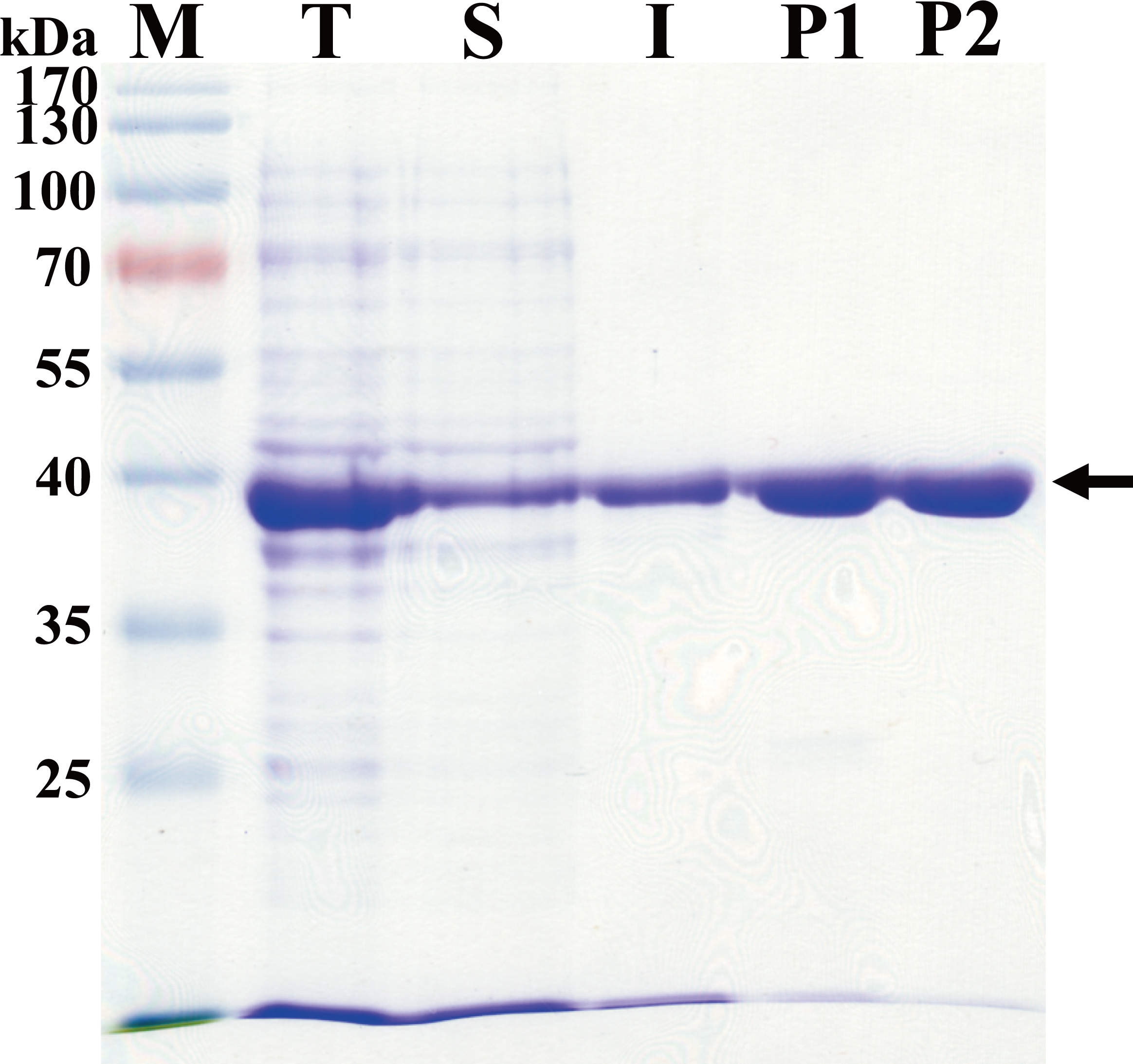


**Figure S2**. SDS-PAGE of the purified EstSTR1 protein. M, Molecular size markers; T, whole-cell extracts; S, soluble fraction; I, insoluble fraction; P1, EstU1 purified by Ni-NTA column; P2, EstU1 purified by Superdex 75 gel filtration column. The purified EstSTR1 corresponded to a molecular mass of approximately 42 kDa and is indicated by arrow.


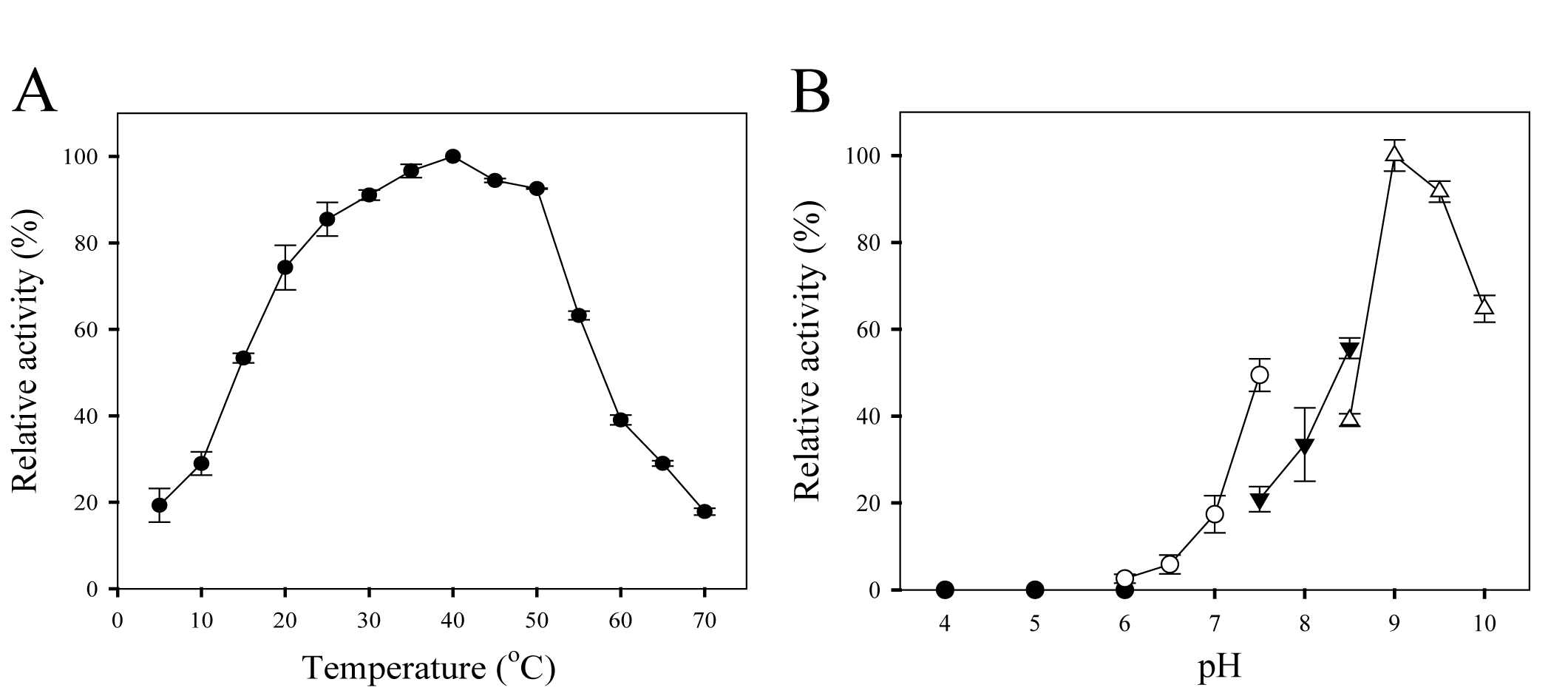


**Figure S3.** Effects of temperature (**A**) and pH (**B**) on the activity of EstSTR1. Enzyme activity was measured using *p*-nitrophenyl butyrate as a substrate at various temperatures. The buffers used were 50 mM sodium acetate buffer (closed circles; pH 4.0 to 6.0), 50 mM sodium phosphate buffer (open circles; pH 6.0 to 7.5), 50 mM Tris–HCl buffer (closed triangles; pH 7.5 to 8.5), and 50 mM CHES buffer (open triangles; pH 8.5 to 10.0). The highest value of each enzyme activity was set at 100%.


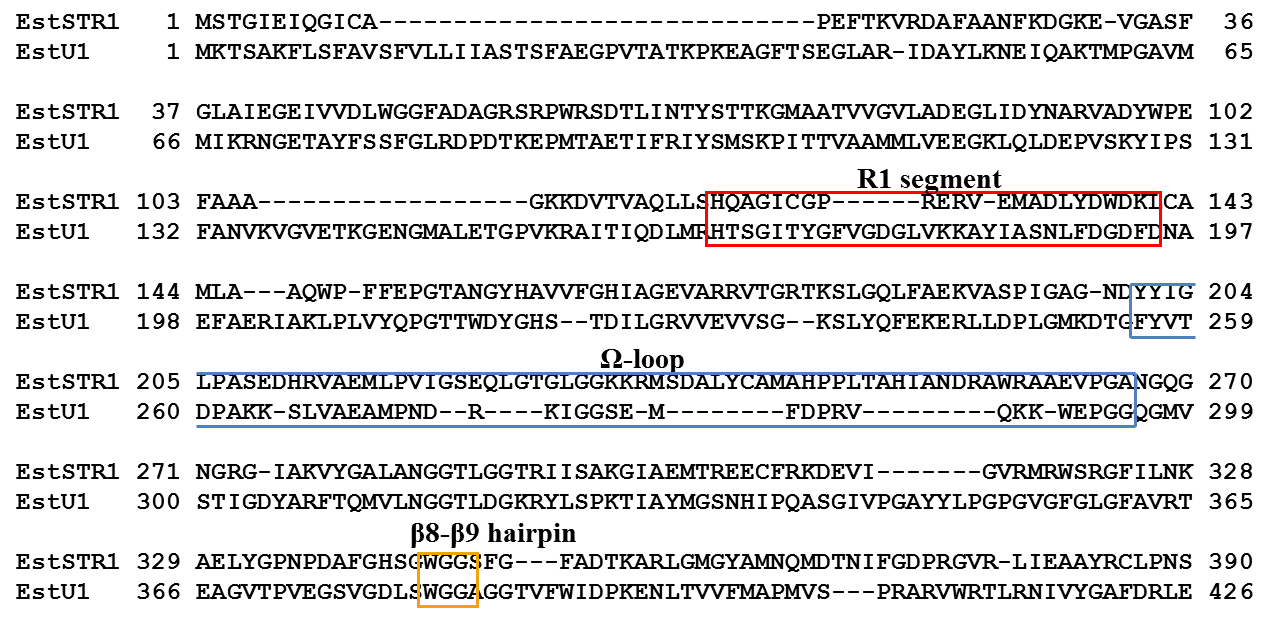


**Figure S4**. Sequence alignment of EstSTR1 with EstU1 which is Family VIII carboxylesterase. R1 segment, Ω-loop, and β8-β9 hairpin regions are indicated with red, blue, and orange boxes, respectively.
